# Treatment of Sarcoma Lung Metastases with Stereotactic Body Radiotherapy

**DOI:** 10.1155/2018/9132359

**Published:** 2018-04-01

**Authors:** Adam D. Lindsay, Edward E. Haupt, Chung M. Chan, Andre R. Spiguel, Mark T. Scarborough, Robert A. Zlotecki, Parker C. Gibbs

**Affiliations:** ^1^Department of Orthopaedic Surgery, University of Connecticut, Farmington, CT, USA; ^2^Department of Orthopaedic Surgery, University of Florida, Gainesville, FL, USA; ^3^Department of Radiation Oncology, University of Florida, Gainesville, FL, USA

## Abstract

**Background:**

The most common site of sarcoma metastasis is the lung. Surgical resection of pulmonary metastases and chemotherapy are treatment options that have been employed, but many patients are poor candidates for these treatments for multiple host or tumor-related reasons. In this group of patients, radiation might provide a less morbid treatment alternative. We sought to evaluate the efficacy of radiotherapy in the treatment of metastatic sarcoma to the lung.

**Methods:**

Stereotactic body radiotherapy (SBRT) was used to treat 117 pulmonary metastases in 44 patients. Patients were followed with serial computed tomography imaging of the chest. The primary endpoint was failure of control of a pulmonary lesion as measured by continued growth. Radiation-associated complications were recorded.

**Results:**

The majority of patients (84%) received a total dose of 50 Gy per metastatic nodule utilizing an image-guided SBRT technique. The median interval follow-up was 14.2 months (range 1.6–98.6 months). Overall survival was 82% at two years and 50% at five years. Of 117 metastatic nodules treated, six nodules showed failure of treatment (95% control rate). Twenty patients (27%) developed new metastatic lesions and underwent further SBRT. The side effects of SBRT included transient radiation pneumonitis (*n*=6), cough (*n*=2), rib fracture (*n*=1), chronic pain (*n*=1), dermatitis (*n*=1), and dyspnea (*n*=1).

**Conclusion:**

Stereotactic body radiotherapy is an effective and safe treatment for the ablation of pulmonary metastasis from sarcoma. Further work is needed to evaluate the optimal role of SBRT relative to surgery or chemotherapy for treatment of metastatic sarcoma.

## 1. Background

Soft tissue and bone sarcomas represent less than 1% of all malignancies diagnosed in the United States annually [1]. The most common site of metastasis is the lung, with pulmonary metastatic disease occurring in 19–50% of patients [2–4]. Metastasectomy and/or chemotherapy are the most common treatments offered to patients with metastatic sarcoma. Pulmonary metastasectomy, either video assisted or through formal thoracotomy, has been shown to increase overall survival in select populations of both osseous and soft tissue sarcoma patients [2, 3, 5–7]. While chemotherapy remains an important tool for treatment of patients with metastatic sarcoma, there is mixed evidence on its survival impact [8–10]. Furthermore, many older patients, as a result of various tumor or comorbidity-related issues, are deemed poor candidates for surgery or chemotherapy.

External beam radiotherapy (EBRT) has been shown to decrease local recurrence when used in conjunction with surgery for primary localized soft tissue sarcoma [11, 12]. The use of SBRT for the treatment of metastatic carcinoma to the lung has been shown to be safe and effective [13]. Previous reports on the use of SBRT for metastatic sarcoma to the lungs exist but are limited by small patient numbers and limited long-term follow-up [14–16]. We sought to evaluate the safety and effectiveness of SBRT for treatment of metastatic sarcoma to the lungs in patients treated at our institution.

## 2. Methods

We performed a retrospective review of 95 patients who had been treated for metastatic sarcoma to the lung between the years 2005 and 2014. Institutional review board approval was obtained prior to chart review. Patients were identified using an institutional database (RadTrac). All patients who had a history of metastatic sarcoma to the lung treated with SBRT were included. We excluded patients who had received prior chest radiation, patients who received whole lung irradiation, patients receiving radiation for a diagnosis other than metastatic sarcoma, those with chest wall metastases, and patients with inadequate follow-up imaging. Fifty-one patients were excluded, leaving 44 patients for review.

Pertinent data including demographic information, primary tumor location, primary tumor histology, primary tumor grade, and previous or concurrent treatment regimens (including surgery and chemotherapy) were collected. All patients were followed with serial computed tomography (CT) of the chest. Treatment failure was defined as any growth of the targeted lesion on subsequent CT imaging. Specific details pertaining to SBRT treatment including dosage, intervals, and associated complications were documented. Social security death index and published obituaries were used to confirm date of death when not formally dictated in the hospital's records.

Target definition for planning radiotherapy treatment of the sarcoma lung metastasis was achieved by performing composite 4D CT imaging during shallow breathing. The image sets were coregistered and incorporated into the Philips Pinnacle treatment planning software system where by an internal tumor volume (ITV) was thus defined. A 5 mm volumetric expansion was then applied to define the planning target volume (PTV). The isodose objective goal for radiotherapy dose delivery was to achieve 95% dose coverage to the ITV, with rapid falloff to an 80% isodose coverage of the PTV. Intensity modulated radiotherapy delivery planning through either step and treat or dynamic arc techniques were typically utilized. Patient positioning was ensured through utilization of Vac-Lok immobilization foam molds and shallow breathing routines. Cone beam CT imaging was performed immediately before SBRT treatment delivery. Image-guided radiotherapy was administered with radiation physicist and physician jointly verifying localization and coverage of each metastatic lesion. All SBRT/IGRT technologies utilized for treatment delivery were performed on Elekta Synergy or infinity radiotherapy treatment delivery systems. Centrally located lesions (within 2 cm of the bronchial tree) were treated in 10 fractions to minimize toxicity, while peripherally located lesions were treated in 5 or less fractions.

Statistical analysis was carried out using EZR statistical software version 1.30 [17]. A Kaplan–Meier estimate was used for survival analysis. Log rank was used to determine difference in survival rate, with a *p* value of <0.05 considered as significant. The Common Terminology Criteria for Adverse Events (CTCAE) version 4.0 was used to grade radiation-associated toxicity [18].

## 3. Results

Stereotactic body radiotherapy was used to treat 117 pulmonary metastases in 44 patients. The median age was 67 (range 19–91). Twenty-two of the patients treated were male. The predominant histology of the primary tumor was undifferentiated pleomorphic sarcoma (UPS)/malignant fibrous histiocytoma (22 patients) (Table 1). The majority of these patients had high-grade tumors (33 of 44, 75%). The median number of pulmonary metastases treated per patient was two (range 1–7). All lesions were treated simultaneously. The median size of lesion treated was 2.1 cm (range 0.8 cm–7 cm). Seventeen patients had prior thoracic surgery for metastatic disease. Chemotherapy was administered to 23 patients. Nine patients received chemotherapy as part of their initial treatment regimen, while 14 received chemotherapy after metastatic disease was diagnosed. The location of the primary tumor was in the extremity in the majority of patients (36 of 44, 82%) (Table 1). Six patients had a primary tumor arising from bone, and the rest were soft tissue sarcomas.

The majority of patients (37 of 44, 84%) received a dose of 50 gray (Gy) per pulmonary nodule. Most commonly, patients received radiation over 10 fractions (32 of 44, 73%).  Table 2 displays a summary of dosing and fraction regimens used.

**Figure 1:**
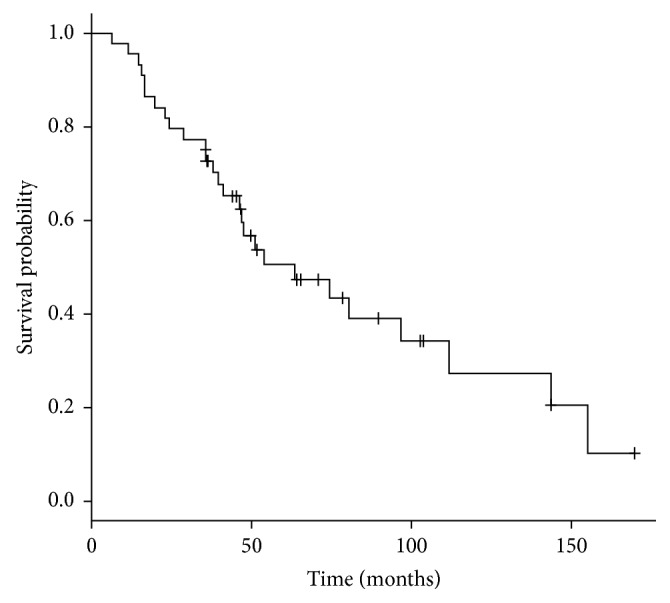
Actuarial overall survival from diagnosis.

**Figure 2:**
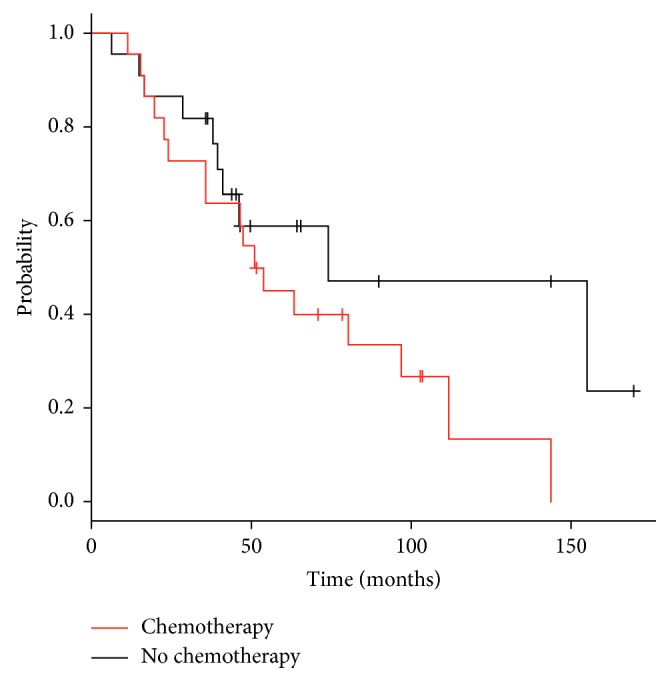
Actuarial overall survival from diagnosis in patients treated with and without chemotherapy.

**Figure 3:**
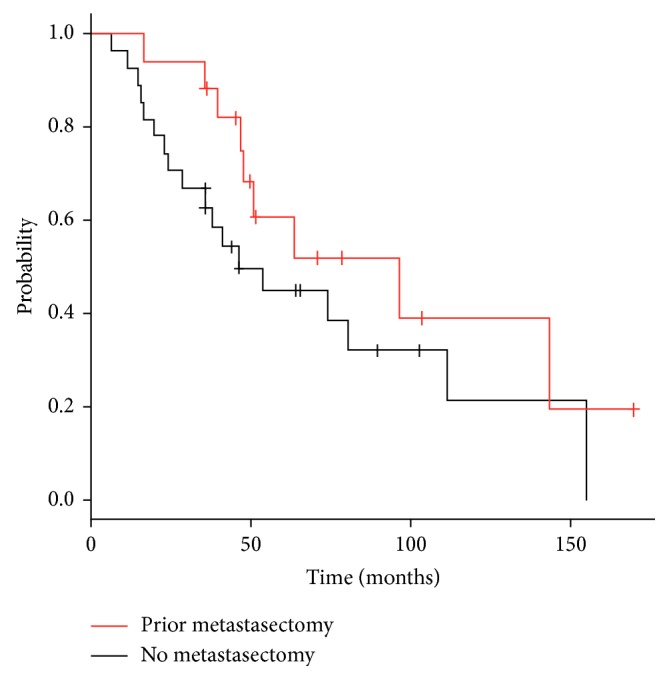
Actuarial overall survival from diagnosis in patients with and without a history of metastasectomy.

The median interval follow-up from initiation of SBRT to death or last imaging follow-up was 14.2 months (range 1.6–98.6 months). At the time of analysis, 27 patients had died (61%). Overall survival was 82% at two years and 50% at five years (Figure 1). Median overall survival was 63.3 months (95% confidence interval 31.0–95.7). Overall survival was not improved in patients previously or concurrently treated with chemotherapy (median survival 52.4 months with chemotherapy versus 74.3 months without chemotherapy, *p* value = 0.161) (Figure 2). While a history of prior metastasectomy did show a trend towards longer survival, this did not reach statistical significance (median survival 96.8 months with prior metastasectomy versus 46.3 months without metastasectomy, *p* value = 0.21) (Figure 3).

**Figure 4:**
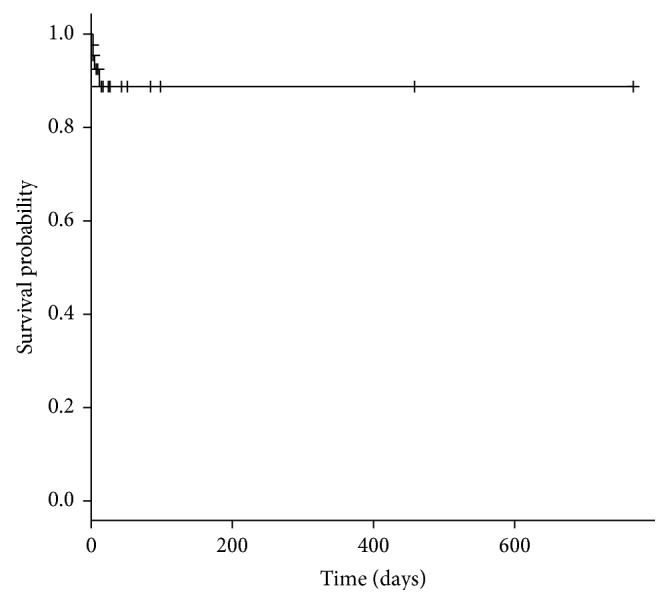
Control of pulmonary nodule growth after stereotactic body radiotherapy (SBRT) treatment.

Four of the forty-four patients (9%) with a total of 6 pulmonary nodules showed progression in size of the pulmonary nodule after treatment with SBRT (Figure 4). This represents a local pulmonary control rate of 95% (6 of 177 pulmonary nodules). The mean time to note nodule growth on CT chest imaging was 4.5 months. Three of the four patients progressed to multiple bilateral pulmonary nodules, while one progressed to multiple pulmonary and extrapulmonary metastases. Two of these patients had metastatic leiomyosarcoma, one had a spindle cell sarcoma not otherwise specified, and one had metastatic undifferentiated pleomorphic sarcoma. The primary tumor was graded as high grade in three of these patients and intermediate grade in one. The median lesion size was 1.8 cm in maximum dimension (range 1.1–4.0 cm). All of these nodules received 50 Gy in 10 fractions.

Further analysis revealed 12 patients (27%) who developed new distinct pulmonary metastases following initial radiation (Table 3). All 12 of these patients had shown no growth after the initial round of SBRT. Twenty new nodules were treated with SBRT in these 12 patients. All of these nodules demonstrated no growth after SBRT treatment. Five of these patients received a third round of SBRT for new pulmonary nodules, again with all patients showing no growth of irradiated pulmonary nodules. The mean time from the end of initial SBRT for pulmonary metastases and the diagnosis of new pulmonary metastases was 9.6 months. In the five patients who received a third round of pulmonary SBRT, the mean time between second and third rounds of SBRT was 23.3 months (Table 3).

Eleven patients (25%) had documented radiation-associated complications (Table 4). The most common complication was a transient pneumonitis that required no formal treatment. Two patients with pneumonitis also developed a chronic cough. All toxicities could be graded as ≤3, except for one patient who developed an esophageal stricture. This patient had been treated for a 3.9 cm right hilar metastasis from an osteosarcoma. He developed esophagitis within one month of completion of SBRT and eventually required parenteral nutrition. Esophagogastroduodenoscopy confirmed the esophageal stricture and balloon dilation was performed.

## 4. Discussion

Improvements in the ability to safely deliver ablative doses of radiation, specifically with techniques such as SBRT, have shown promise for the treatment of several cancer types metastatic to the lung [19]. Navarria et al. showed a 89% local control rate at three years using SBRT to treat a variety of cancer types metastatic to lung, including colorectal and genitourinary carcinomas as well as sarcomas [20]. Baschnagel et al. found similar results with an 85% control rate at three years using SBRT to treat various cancer types metastatic to lung [13]. Two prospective phase-2 studies have shown promising results with the use of SBRT for patient with oligometastatic carcinoma in the lung [21, 22]. SBRT has also shown promise for patients with early stage nonsmall cell lung carcinoma deemed poor operative candidates, with some authors arguing that SBRT is as effective as surgical resection [23, 24].

Traditionally, patients with metastatic sarcoma to the lung have been managed with metastasectomy and chemotherapy alone or in combination. Treasure et al. [25] performed a systematic review of pulmonary metastasectomy for metastatic sarcomas. They reviewed 18 articles and found five-year survival rates averaged 34% and 25% for patients with metastatic osseous and soft tissue sarcoma, respectively. Forty-three percent of patients required subsequent metastasectomy. Numerous studies have examined the efficacy of various chemotherapy regimens in patients with metastatic soft tissue sarcoma. Karavasilis et al. [9] recently published on a series of 488 patients with advanced soft tissue sarcoma treated with first-line chemotherapy. They noted a treatment response in approximately half of these patients, but overall survival at 5 years was only 9%.

We present the results of our institution's use of stereotactic body radiotherapy for the treatment of metastatic sarcoma to the lungs. We found a 95% response rate to SBRT. Overall survival at five years was 50%. The majority of patients tolerated radiation well, with only eleven patients developing radiation-associated complications. We believe this report constitutes the largest cohort of sarcoma patients treated with SBRT that exists in the literature to date. More importantly, our results are commensurate with those previously published in similar patient populations [14–16, 26, 27] (Table 5).

We identified four patients (9%) in our series that did not respond to SBRT. While it is difficult to draw conclusions from this group, it was evident that all four patients had progression not only of the treated lung mass, but also systemically with development of further pulmonary and extrapulmonary disease foci. Also of interest was the subgroup of five patients (11%) who had been treated with three rounds of SBRT for continued progression of pulmonary metastatic disease. All of these patients showed ablation of pulmonary nodules with SBRT and also had an increased time interval between last SBRT treatment and identification of a new pulmonary nodule. In this subset of patients, intermittent treatment with SBRT seems to result in adequate disease control and prolonged survival.

The limitations of this study include its retrospective design, the variable histology types that were included, the use of chemotherapy in approximately half of our patients, and the variation in SBRT fractionation and dosing regimens that were employed. In conclusion, we feel our results show that SBRT is a safe and effective method for treating metastatic sarcoma to the lung. We found no evidence to suggest that chemotherapy improved survival in this patient population. Overall survival in this series was 50% at 5 years, suggesting that SBRT used for local ablation of metastatic sarcoma is associated with good outcomes. Further prospective studies are warranted to determine the efficacy of SBRT as compared to surgery and/or chemotherapy.

## Figures and Tables

**Table 1:** Patient characteristics.

Number of patients	44
Number of lesions treated	117
Age (range)	67 (19–91)
Primary tumor histology	
UPS/MFH	21
Leiomyosarcoma	5
Synovial sarcoma	5
Ewings	2
Hemangiopericytoma	2
Myxofibrosarcoma	2
Spindle cell sarcoma	2
Chondrosarcoma	1
Clear cell chondrosarcoma	1
Dedifferentiated chondrosarcoma	1
Dedifferentiated liposarcoma	1
Osteosarcoma	1
Primary tumor location	
Extremity	36 (82%)
Pelvis	4 (9%)
Other	4 (9%)
Primary tumor grade	
Low	4 (9%)
Intermediate	7 (16%)
High	33 (75%)
Prior chemotherapy	
Yes	23 (52%)
No	21 (48%)
Prior thoracic surgery	
Yes	17 (39%)
No	27 (61%)
Extrathoracic disease	
Yes	16 (36%)
No	28 (64%)
Median number of mets treated (range)	2 (1–7)

**Table 2:** SBRT doses and fractions.

Dose (Gy)/fractions	Pts. (%)
50/10	32 (73%)
50/5	5 (11%)
48/12	2 (5%)
30/6	2 (5%)
55/10	1 (2%)
45/5	1 (2%)
36/6	1 (2%)

**Table 3:** Patients with >1 SBRT treatment.

	Two rounds	Three rounds
Patients	12 pts.	5 pts.
Nodules	20	6
Primary tumor histology		
UPS/MFH	5	3
Myxofibrosarcoma	2	
Leiomyosarcoma	1	
Chondrosarcoma	1	
Hemangiopericytoma	1	1
Spindle cell sarcoma	1	
Synovial sarcoma	1	1
Average time from last SBRT (months)	9.6	23.3
Pulmonary local control	100%	100%

**Table 4:** Radiation-associated side effects.

Pneumonitis	6
Cough	2
Esophageal stricture	1
Pain	1
Dermatitis	1
Rib fracture	1
Dyspnea	1

**Table 5:** Published series using SBRT for sarcoma lung metastases.

Author/year	Patients	Lung nodules treated	Pulmonary control	5-year survival
Dhakal et al. 2012 [14]	14	74	97%	28%
Mehta et al. 2014 [16]	13	25	94%	n/a
Navarria et al. 2015 [20]	28	51	96%	60%
Baumann et al. 2015 [23]	26	32	91%	n/a
Lindsay 2018	44	117	95%	50%
